# An Assessment of Potential Exposure and Risk from Estrogens in Drinking Water

**DOI:** 10.1289/ehp.0900654

**Published:** 2009-10-13

**Authors:** Daniel J. Caldwell, Frank Mastrocco, Edward Nowak, James Johnston, Harry Yekel, Danielle Pfeiffer, Marilyn Hoyt, Beth M. DuPlessie, Paul D. Anderson

**Affiliations:** 1 Johnson & Johnson Worldwide Environment, Health, and Safety, New Brunswick, New Jersey, USA; 2 Pfizer Inc., New York, New York, USA; 3 Johnson & Johnson Pharmaceutical Research and Development, Raritan, New Jersey, USA; 4 Wyeth, Madison, New Jersey; 5 ARCADIS, Chelmsford, Massachusetts, USA; 6 AMEC Earth & Environmental, Westford, Massachusetts, USA; 7 Department of Geography and Environment, Boston University, Boston, Massachusetts, USA

**Keywords:** acceptable daily intake, dietary intake, drinking water, environmental sources, estrogen, excretion, PhATE, phytoestrogen, surface water

## Abstract

**Background:**

Detection of estrogens in the environment has raised concerns in recent years because of their potential to affect both wildlife and humans.

**Objectives:**

We compared exposures to prescribed and naturally occurring estrogens in drinking water to exposures to naturally occurring background levels of estrogens in the diet of children and adults and to four independently derived acceptable daily intakes (ADIs) to determine whether drinking water intakes are larger or smaller than dietary intake or ADIs.

**Methods:**

We used the Pharmaceutical Assessment and Transport Evaluation (P*h*ATE) model to predict concentrations of estrogens potentially present in drinking water. Predicted drinking water concentrations were combined with default water intake rates to estimate drinking water exposures. Predicted drinking water intakes were compared to dietary intakes and also to ADIs. We present comparisons for individual estrogens as well as combined estrogens.

**Results:**

In the analysis we estimated that a child’s exposures to individual prescribed estrogens in drinking water are 730–480,000 times lower (depending upon estrogen type) than exposure to background levels of naturally occurring estrogens in milk. A child’s exposure to total estrogens in drinking water (prescribed and naturally occurring) is about 150 times lower than exposure from milk. Adult margins of exposure (MOEs) based on total dietary exposure are about 2 times smaller than those for children. Margins of safety (MOSs) for an adult’s exposure to total prescribed estrogens in drinking water vary from about 135 to > 17,000, depending on ADI. MOSs for exposure to total estrogens in drinking water are about 2 times lower than MOSs for prescribed estrogens. Depending on the ADI that is used, MOSs for young children range from 28 to 5,120 for total estrogens (including both prescribed and naturally occurring sources) in drinking water.

**Conclusions:**

The consistently large MOEs and MOSs strongly suggest that prescribed and total estrogens that may potentially be present in drinking water in the United States are not causing adverse effects in U.S. residents, including sensitive subpopulations.

Detection of estrogens in the environment has raised concerns in recent years because of the potential of these compounds to affect both wildlife and humans. The incomplete removal by publicly owned treatment works (POTWs) of excreted endogenous estrogens and prescribed estrogens leads to their introduction into surface waters and potentially into drinking water sources that rely on surface water. Estrogens, specifically estrone (E1), 17β-estradiol (E2), estriol (E3), and ethinyl estradiol (EE2), have been detected in numerous studies of wastewater influents and effluents (Baronti et al. 2000; Belfroid et al. 1999; Desbrow et al. 1998; Ferguson et al. 2001; Heberer 2002; Huang and Sedlak 2001; Huggett et al. 2003; Laganà et al. 2000; Mouatassim-Souali et al. 2003; Nasu et al. 2001; Rodgers-Gray et al. 2000; Spengler et al. 2001; Ternes et al. 1999a, 1999b, 2002), and their presence has been confirmed in U.S. and European surface waters (Aherne and Briggs 1989; Belfroid et al. 1999; Heberer 2002; Kolodziej et al. 2003; Kolpin et al. 2002; Kuch and Ballschmiter 2001). More recently, several estrogens have also been detected in the source water of drinking water treatment plants but not in the finished water (Benotti et al. 2009).

The effects of estrogens on fish and other aquatic organisms have been widely studied (see Caldwell et al. 2008). However, fewer studies have evaluated the potential effects of estrogens in surface water on humans. Moreover, the available studies on exogenous estrogens reach differing conclusions on potential human effects (Aherne and Briggs 1989; Andersson and Skakkebaek 1999; Christensen 1998; Webb et al. 2003). Based on independent worst-case exposure estimates, Aherne and Briggs (1989) and Christensen (1998) concluded that risks from environmental sources of the synthetic hormone EE2 were negligible compared with normal body concentrations of estrogens. Webb et al. (2003) noted that worst-case indirect exposure to EE2 via drinking water would be three to four orders of magnitude lower than endogenous production rates of E2. Andersson and Skakkebaek (1999), however, raised concern that there may be no threshold level regarding the action of exogenous estrogens, particularly for prepubertal males. Previous reports (Fritsche and Steinhart 1999; Hartmann et al. 1998) concluded that dietary intake of the endogenous estrogens is minimal compared with human production rates.

For the most part, articles reporting detection of estrogens in surface and drinking waters both in the public press (Donn et al. 2008a, 2008b, 2008c) and in the scientific literature (reviewed by Ying et al. 2002), provide little context as to whether potential drinking water exposures are large or small compared with other sources of exposure (e.g., dietary intake) or compared with acceptable daily intakes (ADIs). This makes it difficult to determine whether exposures from drinking water derived from surface water are a significant source of overall estrogen exposure or have the potential to exceed ADIs and, thus, whether they deserve additional evaluation.

The analysis presented here is, to our knowledge, the first exposure assessment for estrogens in drinking water that distinguishes among the potential sources of estrogens. In an earlier human health risk assessment of pharmaceuticals in U.S. surface waters, Schwab et al. (2005) found no appreciable health risk from 26 drugs representing 14 different drug classes, but estrogens were not included in the study. In the present study we used a weight-of-evidence approach to determine whether predicted exposure to trace levels of prescribed and naturally occurring estrogens in drinking water has the potential to cause effects. We developed several lines of evidence which fall into two general categories.

The first category consists of comparing a typical U.S. resident’s potential drinking water exposures with background dietary exposures (e.g., exposure via consumption of milk or in the overall diet). Naturally occurring, animal-derived estrogens (e.g., E1, E2, and E3) have been measured in a wide variety of foods that are regularly consumed by most Americans (Doyle 2000; Fritsche and Steinhart 1999; Hartmann et al. 1998; Henricks et al. 1983; Tsujioka et al. 1992). Dietary intake is likely the dominant pathway of estrogen exposure for most people in the United States of both sexes and all ages [except women using prescribed estrogens for birth control, hormone therapy, or hormone replacement therapy) (Fritsche and Steinhart 1999)]. Although we do not know whether dietary exposures are or are not associated with effects (adverse or beneficial), we do know they represent a consistent daily exposure for U.S. residents. Whether drinking water exposures are large or small compared with dietary exposure to these same estrogens provides important perspective as to their relative significance.

The second category of lines of evidence consists of comparing drinking water exposures with toxicity-based benchmarks assumed to be without adverse effect [e.g., the World Health Organization (WHO) ADI, the threshold for toxicologic concern (TTC), occupational exposure limits, and *Australian Guidelines for Water Recycling* [Environment Protection and Heritage Council (EPHC) et al. 2008)]. These benchmarks represent estimates of exposure that are assumed to be safe and are derived using commonly accepted public health practices. Whether drinking water exposures are larger or smaller than these benchmarks indicates whether they may or may not be associated with adverse effects.

## Methods

Estimating exposure to estrogens via drinking water requires information about the concentrations of estrogens in drinking water and the amount of water consumed by a typical person in the United States. The U.S. Environmental Protection Agency (EPA) recommended water ingestion rates of 0.87 L/day for children and 1.4 L/day for adults were used to estimate drinking water consumption (U.S. EPA 1997). Predicted concentrations in drinking water were used instead of measured concentrations because few studies have measured estrogen concentrations in U.S. drinking water, and those that are available report primarily nondetected concentrations [see Supplemental Material, available online (doi:10.1289/ehp.0900654.S1 via http://dx.doi.org/); see also Hannah et al. 2009]. The predicted environmental concentrations (PECs) of synthetic estrogens and endogenous estrogens in drinking water resulting from human use and excretion were estimated using the P*h*ATE (Pharmaceutical Assessment and Transport Evaluation) model, version 2.1.1 (Anderson et al. 2004). P*h*ATE requires several compound-specific inputs, including the per capita use or excretion rate, metabolism, POTW removal rate, in-stream removal, and drinking water treatment removal. Removal rates were not available for all estrogens. When no removal information was available for a particular estrogen and a particular removal mechanism, we assumed that no removal occurred to assure that drinking water PECs were not underestimated. A summary of P*h*ATE inputs is provided in the Supplemental Material, Tables SM-4 through SM-9 (doi:10.1289/ehp.0900654.S1).

P*h*ATE generates PECs for approximately 2,710 stream segments in 11 U.S. watersheds and for 184 drinking water treatment plants serving approximately 10,800,000 people located on the modeled stream segments. The PECs used in this analysis are those for each of the drinking water treatment systems included in P*h*ATE. P*h*ATE is able to generate PECs for both mean flow conditions and 7Q10 low-flow (i.e., the lowest consecutive 7-day low flow that occurs on average once every 10 years) conditions. To be conservative, in this analysis we used 7Q10 low-flow PECs because this is when estrogen concentrations in surface water used as source water for a drinking water treatment plant would be at their highest (as opposed to annual average flow when concentrations would be lower).

PECs generated by P*h*ATE are grouped five ways to enable discrimination among different sources of estrogens in drinking water and among types of estrogens. Potential exposures are presented for prescribed synthetic estrogen alone (i.e., EE2), prescribed endogenous estrogens (i.e., E1, E2, and E3 prescribed for therapeutic use), total prescribed estrogens (i.e., E1, E2, E3, and EE2 prescribed for therapeutic use), naturally occurring endogenous estrogens (i.e., naturally occurring animal-derived E1, E2, and E3), and total estrogens (E1, E2, E3, and EE2 from all sources). For dietary and ADI comparisons that required combining exposure to individual estrogens, total estrogen exposure was expressed as estradiol equivalents (E2-eq), which were estimated based on alpha receptor binding for E1, E2, and E3 (assumed to have relative biological activity of 0.1, 1.0, and 0.038, respectively). To avoid potentially underestimating the estrogenic activity of synthetic estrogen, EE2 was assumed to have 10 times the activity of E2 (i.e., a relative potency of 10). More detailed discussion of the relative biological activity adjustments is presented in Supplemental Material (doi:10.1289/ehp.0900654.S1).

### Dietary benchmarks

Estrogens have specific direct and indirect effects that may be functions of age as well as sex (Aksglaede et al. 2006; Andersson and Skakkebaek 1999; Dey et al. 2000). Some potentially sensitive subpopulations include prepubescent males who have low natural serum estrogen levels and an expected low metabolic clearance rate (Andersson and Skakkebaek 1999; Klein et al. 1996), postmenopausal women with naturally low estrogen levels, and people with specific dietary preferences, such as vegetarians, that increase their exposure to phytoestrogens. We examined the differences in total intake of estrogens among several different age groups, between males and females, and among dietary preferences. Women taking prescribed estrogens for therapeutic use had the largest total daily exposure to estrogens, followed by infants fed soy-based formula, then by infants on breast milk or milk-based formula, and last by children and adults (data not shown).

Given the concern about exposure of prepubescent males (an assumed sensitive subpopulation) to estrogens in drinking water, this analysis used estrogen exposure from milk consumption of young children as one set of dietary benchmarks. The second set of dietary benchmarks is the daily exposure of an adult female eating an omnivorous diet. This is likely to be representative of the daily estrogen exposure for the majority of adults in the United States.

We estimated dietary exposure to endogenous estrogens by combining the reported concentrations of estrogens in foodstuffs with the average consumption rate of the foodstuff [see Supplemental Material, Tables SM-1 through SM-3 (doi:10.1289/ehp.0900654.S1)]. Endogenous estrogen intake is likely biased low because concentration data for one or more estrogens in a particular food may be absent and data for other foodstuffs expected to contain estrogens have not been published. Additionally, this analysis does not include the contribution of phytoestrogens to the background estrogenic activity present in a typical U.S. diet. Given that adult premenopausal women are reported to have a total isoflavone intake of 1.78 mg/day (Horn-Ross et al. 2002) and 2.17 mg/day (Huang et al. 2000) and a total lignan intake of 0.108 mg/day (Horn-Ross et al. 2002), 0.525 mg/day (McCann et al. 2003), and 0.645 mg/day (deKleijn et al. 2002), omission of phytoestrogens understates background dietary estrogen exposure. Although biologically active (Masutomi et al. 2004; Montani et al. 2008; Rimoldi et al. 2007), phytoestrogens were excluded because data on their concentrations in many foods are not available and their estrogenic potency relative to the animal-derived endogenous estrogens remains difficult to quantify.

### Toxicity-based benchmarks

In addition to comparing estrogen exposure via drinking water with background dietary exposure, this analysis compares exposure to estrogens in drinking water with four independently derived ADIs (or sets of ADIs) available in the literature.

The WHO (2000) derived an ADI of 0.05 μg E2/kg body weight (BW)/day based on a no observed effect level (NOEL) of 0.3 mg E2/person/day associated with changes in several hormone-dependent parameters. The WHO divided the NOEL by an uncertainty factor (UF) of 10 to account for “normal variation” among individuals and a second UF of 10 to account for “sensitive populations.” In the United States, the assumed adult body weight is 60 kg, so the adjusted WHO whole-body ADI is 3 μg E2/person/day.

The TTC is an acceptable daily exposure presented by Kroes et al. (2004) based on a review of toxicity data from a variety of chemicals. In the present study, we conservatively assumed that estrogens are structurally active compounds and evaluated them using the TTC of 0.15 μg/person/day derived by Kroes et al. (2004) for compounds with a structural alert. Such compounds were assigned the lowest TTC. Had a higher (i.e., less conservative) TTC been used, the MOS estimated in this analysis would have been higher.

Because EE2 is produced in a manufacturing environment, its makers developed occupational exposure limits for the protection of workers using all available toxicologic and pharmacologic data to protect against potential hazards, primarily from dust inhalation. The myriad biologic responses to estrogen exposure argue for use of such an integrated assessment of effect in risk assessment. The occupational exposure limit is established to protect humans against all biologically significant effects and is based on *in vivo* studies and human experience. The EE2 occupational exposure limit of 0.01 μg/m^3^ used in our analysis is the most recent and lowest of five occupational exposure limits developed by different manufacturers (Johnson & Johnson, unpublished data). It was converted to an allowable dose of 0.07 μg EE2/person/day by adjusting from an allowable air concentration to an ADI by multiplying by an assumed inhalation rate of 10 m^3^/person/day and multiplying by 5/7 to account for the difference in number of days a worker is exposed per week versus a member of the general public. An additional 10-fold reduction to account for sensitive populations, in this case potential effects on the developing infant, results in an ADI of 0.007 μg EE2/person/day. Similarly, ADIs of 0.07, 0.02, and 0.07 μg/person/day for E1, E2, and E3, respectively, were derived from their respective occupational exposure limits [0.1 μg/m^3^, 0.029 μg/m^3^, and 0.1 μg/m^3^ (Caldwell DJ, personal communication; Johnson & Johnson, unpublished data)] using the same approach as presented for EE2.

Australia developed water reuse guidelines for estrogens (EPHC et al. 2008). Australia used the WHO ADI to develop the E2 guideline, however; the ADIs for E1, E3, and EE2 were derived by applying a 10,000-fold safety factor to the lowest therapeutic dose, including a safety factor of 10 to account for sensitive populations. The resulting ADIs for E1, E2, E3, and EE2 are 0.052, 3, 0.084, and 0.0026 μg/person/day, respectively.

## Results

### PECs generated by PhATE

P*h*ATE’s ability to predict representative surface water concentrations has been documented previously for a variety of compounds (Anderson et al. 2004) and more recently for EE2 in a critical review comparing surface water PECs with all available measured concentrations of EE2 in surface water (Hannah et al. 2009). Drinking water PECs are slightly lower than surface water PECs because drinking water intakes are present on < 10% of stream segments, and these segments are unlikely to be immediately downstream of POTWs. Segments immediately downstream of POTWs have the highest surface water PECs.

**Figure 1:**
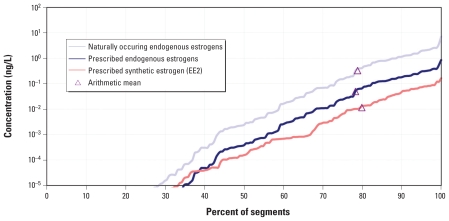
Cumulative distribution (and arithmetic mean) of PECs generated by P*h*ATE for three different categories of estrogens in U.S. drinking water assuming critical low-flow conditions (7Q10). For the endogenous estrogens, the combined concentrations of E1, E2, and E3 were not adjusted for differences in biological activity.

The P*h*ATE model is able to generate PECs associated with various sources of estrogens to drinking water. The ability to distinguish the relative contribution of different sources points out a unique benefit of modeling concentrations because it is not possible through measurement, for example, to distinguish prescribed E2 from naturally occurring E2 in a water sample. Excreted naturally occurring endogenous estrogens have the highest drinking water PECs, followed by prescribed endogenous estrogens. Prescribed synthetic estrogens (i.e., EE2) have the lowest PECs (Table 1, Figure 1).

We estimated drinking water exposures using the arithmetic mean of drinking water PECs assuming 7Q10 low-flow conditions (Table 1, Figure 1). The arithmetic mean low-flow PEC represents the 79th, 78th, and 80th percentile of the cumulative drinking water system PECs for naturally occurring endogenous, prescribed endogenous, and prescribed synthetic estrogens, respectively. Use of the arithmetic mean low-flow PEC leads to conservative but not extreme estimates of potential drinking water exposure and is consistent with the use of mean, rather than upper bound or maximum, concentrations of endogenous estrogens in foodstuffs. Most of the time, concentrations in drinking water will be lower because actual flow will be higher.

### Comparison of drinking water to dietary exposures

We present two sets of dietary comparisons. To address concerns about the potential exposures of preadolescent children to estrogens predicted to be in drinking water, in the first set we compared a young child’s exposure to estrogens in drinking water with his or her dietary exposure to naturally occurring estrogens in milk (milk consumption is encouraged in young children) (Department of Health and Human Services 2005). To evaluate exposure of the general population, we compared an adult’s predicted exposure to estrogens via drinking water with an omnivore’s exposure to naturally occurring estrogens in the overall diet.

**Figure 2:**
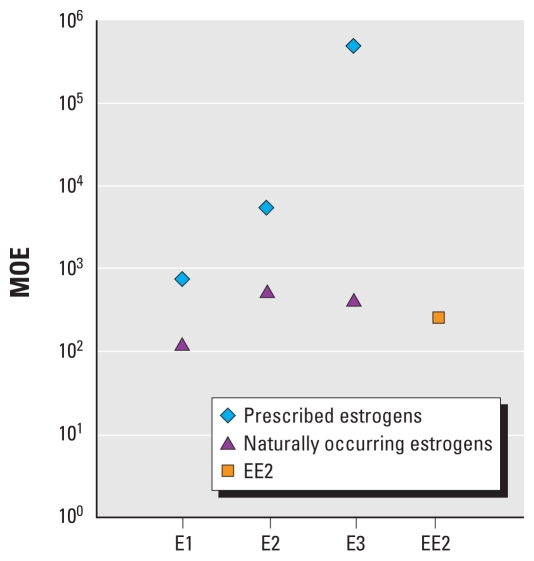
MOEs (equal to the predicted estrogen intake from milk divided by the predicted estrogen intake from drinking water) for a young child. For E1, E2, and E3, MOEs are shown for exposure to prescribed estrogens predicted to be in drinking water and for naturally occurring estrogens predicted to be in drinking water. A single MOE is shown for EE2 because the only source of EE2 in drinking water is assumed to be therapeutic use (i.e., prescribed). MOEs for E1, E2, and E3 are based on the mass-based concentration of each estrogen in drinking water and milk. The EE2 MOE is based on the E2-eq concentration of EE2 in drinking water and of E1, E2, and E3 combined in milk.

The results of the comparison of drinking water to dietary estrogen exposures are referred to as margins of exposure (MOEs). For the young child, we present MOEs for individual estrogens as well as all estrogens combined. Presenting MOEs on an individual estrogen basis allows for the derivation of MOEs without using relative potency adjustments for the endogenous estrogens, thus eliminating the uncertainty associated with such adjustment factors. A young child’s exposure to naturally occurring E1, E2, and E3 through typical consumption of milk [assumed to be about 0.42 L/day; see Supplemental Material (doi:10.1289/ehp.0900654.S1)] is approximately 730, 5,000, and 480,000 times, respectively, greater than his or her exposure to trace concentrations of those estrogens predicted to be in drinking water as a result of human therapeutic use (Figure 2). The MOEs for naturally occurring endogenous estrogens in drinking water (ranging from approximately 100 to 600; Figure 2) are smaller than the MOEs for prescribed estrogens, indicating that natural sources of E1, E2, and E3 contribute more to drinking water exposures than do prescribed sources.

Because EE2 does not occur naturally in milk, we compared a child’s predicted exposure to EE2 via drinking water (expressed as E2-eq) with the E2-eq concentration in milk. The EE2 drinking water MOE is about 250 (i.e., a child’s exposure to EE2 in drinking water is about 250-fold smaller than his or her E2-eq exposure from drinking milk; Figure 2). Even when all sources of estrogens in drinking water are considered, the E2-eq exposure from drinking water for a young child is about 150 times lower than the exposure from milk alone (Table 2).

Comparison of an adult’s potential exposure to estrogens via drinking water with overall dietary intake of E1, E2, and E3 reveals that the MOEs for predicted drinking water intake vary from 82 to 1,700 depending on estrogen category (Table 2). Prescribed endogenous estrogens have the largest MOE (1,700), followed by naturally occurring endogenous estrogens (MOE = 220), prescribed synthetic estrogens (MOE = 140), total prescribed (MOE = 130), and finally, total estrogens (MOE = 82; Table 2).

### Comparison of drinking water exposures to toxicity-based benchmarks

**Figure 3:**
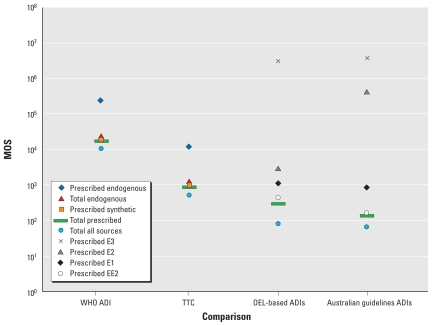
MOSs for adult exposure to estrogens via drinking water for the WHO ADI, the TTC, four ADIs derived from OELs, and the four ADIs used to derive the Australian guidelines (EPHC et al. 2008). For the WHO ADI and the TTC, five MOSs are presented corresponding to five categorizations of estrogens predicted to be in drinking water. MOSs for the WHO ADI and TTC are based on estrogen intakes expressed as E2-eq (i.e., are activity adjusted), as are the MOSs for total prescribed and total all sources comparisons to OELs and Australian guidelines, whereas MOSs for the individual estrogens for OEL and Australian guideline comparisons are based on estrogen intakes expressed on a mass basis (i.e., are not activity adjusted because the estrogen-specific ADIs embody differences in activity).

The results of the comparisons of drinking water estrogen intake to toxicity-based benchmarks are referred to as margins of safety (MOSs), which provide an estimate of how many times smaller the predicted drinking water intake is than the toxicity-based benchmark. When an adult’s potential prescribed estrogen exposures (expressed as E2-eq) are compared with the WHO ADI of 3 μg E2/person/day, the MOSs are about 18,600, 228,000, and 17,000 for synthetic prescribed, prescribed endogenous, and total prescribed estrogens, respectively (Figure 3). Similarly, when the potential exposures are compared with the TTC of 0.15 μg/person/day, MOSs are about 930, 11,400, and 860 for synthetic, prescribed endogenous, and total prescribed estrogens, respectively (Figure 3). The MOS for naturally occurring estrogens (referred to as “Total endogenous” in Figure 3) is about two times higher than that for prescribed synthetic estrogens, and the MOS for total estrogen exposure from drinking water is about 1.5 times smaller than that for the total prescribed estrogen MOS (Figure 3). When the potential drinking water exposures to prescribed E1, E2, E3, and EE2 are compared with their respective occupational exposure limit–derived ADIs, the MOSs for adults are equal to about 1,150, 3,070, 3,200,000, and 441, respectively, and the combined MOS is 289 (Figure 3). The adult MOSs for prescribed E1, E2, E3, and EE2 in drinking water based on the Australian guidelines (EPHC et al. 2008) are about 840, 440,000, 3,700,000, and 160, respectively, and the combined MOS is 135 (Figure 3). MOSs for young children are approximately two times smaller than those for adults because young children are assumed to consume about two times more water on a per kilogram basis than do adults. The lowest MOSs for children result from comparing total prescribed estrogens (MOS = 55) and total of all estrogens combined (prescribed and naturally occurring, MOS = 28) in drinking water with ADIs derived from the Australian guidelines.

The WHO ADI is derived by applying commonly used UFs to therapeutic doses given to postmenopausal women. Although one of these UFs was intended to account for sensitive individuals, is it possible that a UF of 10 may not fully account for differences in sensitivity between postmenopausal women and either men/boys or children? Recent multigenerational studies have examined the effects of exposure to either E2 or EE2 during gestation and early life stages on the reproductive system of young male rodents (Howdeshell et al. 2008; Latendresse et al. 2009; Tyl et al. 2008). Howdeshell et al. (2008) reported a NOEL of 1.5 μg EE2/kg BW/day, equal to 90 μg EE2/person/day, and Tyl et al. (2008) reported a NOEL of 1 μg E2/kg BW/day, equal to 60 μg E2/person/day, assuming a body weight of 60 kg. Latendresse et al. (2009) summarized results from National Toxicology Program (NTP) studies of chronic and multigenerational reproductive effects of EE2. These authors observed an overall no observed adverse effect level for reproductive effects of 0.7 μg/kg/day in the five-generation reproduction study. Both the chronic and reproductive studies summarized by Latendresse et. al. (2009) reported male mammary gland hyperplasia. The increase seen in the low-dose group (0.1 μg/kg/day) of the reproduction study was marginal in the F_1_ generation, and there was no difference from controls in the F_0_, F_2_, F_3_, F_4_, or F_5_ generations. There was no clear evidence that mammary gland hyperplasia progressed to neoplasia, nor was there amplification of mammary hyperplasia in the continuously exposed F_2_ progeny (i.e., offspring of the F_1_ generation that also were continuously exposed). This indicates there was no carryover of effect from generation to generation (Latendresse et al. 2009). Thus, an overall NOEL based on an endocrine-sensitive end point is 0.1 μg/kg/day for males continuously exposed from the time the F_0_ generation was 6 weeks of age through weaning of the F_3_ generation.

If one were to apply UFs of 10 each for interspecies and intraspecies extrapolation and sensitive populations (total UF of 1,000), the resulting ADIs would be 0.09 μg EE2/person/day and 0.06 μg E2/person/day, respectively, for Tyl et al. (2008) and Howdeshell et al. (2008). We conclude that the study by Latendresse et al. (2009) does not require the additional UF for sensitive populations because it was conducted on that population, and the ADI would be 0.06 μg EE2/person/day. These ADIs fall within the range of the other toxicity benchmarks used in the present study. These recent studies suggest that large MOSs (> 25) would also exist when potential exposures to prescribed estrogens in drinking water are compared with ADIs derived specifically to be protective of reproductive effects in young males. Based on Latendresse et al. (2009) reporting an NOEL for an endocrine-sensitive end point in a potentially sensitive population exposed continuously over multiple generations, application of an additional UF of 10 is protective. Thus, the use of a single UF of 10 to account for sensitive populations is a conservative practice.

## Discussion

The large MOEs that result when predicted drinking water exposures of adults and young children are compared with dietary exposures is an important finding. Although we do not know whether dietary exposures to estrogens are or are not associated with effects (adverse or beneficial), we do know they represent a consistent daily exposure for U.S. residents. Documenting that the potential exposure to total estrogens (prescribed and naturally occurring) in drinking water is at least 82 times lower than our natural background dietary exposure suggests that exposures to estrogens (prescribed or naturally occurring) in drinking water are inconsequential and should have no effect. Indeed, it is likely that naturally occurring day-to-day variation in dietary intake (e.g., having a glass of milk or some cheese one day and not the next) will lead to much larger variations in estrogen exposure than is associated with drinking water intake of prescribed estrogens or naturally occurring estrogens.

Similarly, the large MOSs that result when predicted drinking water exposures are compared with several toxicity-based benchmarks are also an important finding and indicate that predicted drinking water exposures to estrogens are not expected to be associated with adverse effects in the U.S. population. Although there is no consistent approach for applying safety factors for infants and children or other sensitive subgroups, the combined safety factor applied to the toxicity-based benchmarks for intraspecies variability and protection of sensitive subgroups adequately addresses issues associated with potential exposure of the developing fetus, infants, and children. Application of an additional safety factor of 10, as done in this analysis, is a conservative approach, as borne out by Latendresse et al. (2009) in the summary of the NTP studies. It is also consistent with the Food Quality Protection Act of 1996, which applies a default safety factor of 10 for sensitive subpopulations when dealing with pesticides in food products. Given that some of the ADIs we used in the present analysis are derived to be protective of sensitive subpopulations, such as prepubescent boys, the conclusion of an absence of an effect should extend to all segments of the U.S. population.

Even though the MOEs and MOSs resulting from all of the comparisons of estrogens in drinking water are consistently large, the question remains whether the uncertainty associated with the present analysis could lead to a substantially lower MOE or MOS. An underestimate of the MOE and MOS associated with prescribed and naturally occurring endogenous estrogens seems unlikely for several reasons. Perhaps most important is that we did not account for potential removal of estrogens by drinking water treatment plants. Few studies have examined such removal. However, in a recent study Benotti et al. (2009) reported drinking water treatment system removal rates of at least 80% and as much as 99% for E1, E2, and EE2 (E3 removal was not measured). In the present study, incorporating these removal data would have reduced predicted drinking water exposures (and, therefore, increased MOEs and MOSs) by at least 5-fold and likely more. MOEs derived from the dietary comparisons would also have been larger if concentrations of naturally occurring endogenous estrogens were available for all the foods in our diet and if the contribution of phytoestrogens to the estrogen content in food had been included.

MOEs and MOSs may be overestimated for some drinking water systems. The arithmetic mean is equal to approximately the 80th percentile of the distribution of drinking water PECs (Figure 1, Table 1). That means approximately 20% of drinking water systems included in P*h*ATE have critical low-flow PECs greater than those used in this analysis. The maximum critical low-flow drinking water PEC is about 10 times greater than the arithmetic mean PEC (Figure 1). The MOEs and MOSs corresponding to the maximum PEC would be about 10 times smaller than those associated with the mean PEC used in this analysis. However, even if we use the maximum PEC generated by P*h*ATE, prescribed estrogens predicted to be in drinking water still represent a fraction of background dietary estrogen exposure and remain below ADIs; thus, adverse effects are not expected.

MOEs and MOSs for estrogen exposure from all sources in drinking water may also be reduced in situations where drinking water system source water contains endogenous hormones from upstream animal husbandry operations or from pharmaceutical manufacturing. Such operations are not included in P*h*ATE because information on the location and magnitude of such sources is not readily available for most watersheds. The effect on most drinking water PECs of not including animal husbandry sources is expected to be small, given that most drinking water systems are likely to be near populated areas of watersheds and such areas are less likely to have animal husbandry operations than more remote areas of the watersheds. The effect on most drinking water PECs of not including pharmaceutical manufacturing sources is also expected to be small given that most pharmaceutical manufacturing sources provide onsite wastewater treatment and/or discharge to POTWs.

MOEs and MOSs for total estrogenic activity in drinking water may also be lower than shown by this analysis because compounds other than the endogenous and prescribed hormones that are the focus of this study may be present in POTW effluents. A comprehensive assessment of the potential effect of these other compounds is beyond the scope of this analysis. However, several studies investigating the overall estrogenic activity of POTW effluents report that most estrogenic activity is attributable to E1, E2, E3, and EE2 (Aerni et al. 2004; Desbrow et al. 1998; Houtman et al. 2007; Salste et al. 2007). These results suggest that even if the estrogenic potential of the other compounds present in POTW effluents were included, the MOEs and MOSs estimated by this analysis would decrease by no more than about 2-fold.

One potential group of people who may have lower surface-water–related MOEs and MOSs than estimated here are anglers who consume self-caught freshwater fish. A bioconcentration factor of 635 has been reported for EE2 (Länge et al. 2001). Assuming that rate of bioaccumulation is representative of EE2 bioaccumulation in natural waters, an angler consuming 6.5 g of fish from the same surface water that serves as the source of his or her drinking water supply is exposed to as much EE2 through the consumption of fish as is present in 4.1 L of water, or about 2.25 times more daily exposure than he or she gets from drinking water. Although this does not alter the conclusions of this analysis because the MOEs and MOSs remain very large, it does indicate that, for some people, potential exposure to estrogens via consumption of fish may result in greater exposure than from consumption of drinking water.

## Conclusion

The large MOEs and MOSs determined in the present study appear robust and are more likely to understate than overstate actual MOEs and MOSs. The dietary comparison indicates that potential exposures to trace levels of total estrogens (whether from a prescribed or naturally occurring source) predicted to be in drinking water in the United States are at least 82 times lower than exposures from background concentrations of naturally occurring estrogens in the diet. Drinking water exposures are also at least 28 times less than ADIs developed to be protective of sensitive populations. Taken together, the finding of consistently large MOEs and MOSs across all lines of evidence strongly suggests that concentrations of estrogens (including prescribed estrogens) predicted by P*h*ATE to potentially be in drinking water are not causing adverse effects in U.S. residents, including sensitive subpopulations.

## Correction

In the original manuscript published online, some exposure estimates and margins of safety were incorrect. They have been corrected here.

## Figures and Tables

**Table 1:** Summary of PECs for three categories of estrogens in U.S. drinking water.

	PEC (ng/L)			
	90th percentile		Average	
Category, compound	Mean flow	Low flow	Mean flow	Low flow
Endogenous estrogens from diet and naturally produced				
E1	0.1	1.1	0.03	0.26
E2	0.02	0.19	0.01	0.05
E3	0.02	0.02	0.01	0.02
Prescribed endogenous estrogens				
E1	0.02	0.18	0.01	0.04
E2	0.002	0.02	0.0006	0.005
E3	0.000015	0.000013	0.000006	0.000016
Prescribed synthetic estrogens				
EE2	0.003	0.05	0.001	0.01

**Table 2:** MOEs for a child and an adult.

	Child		Adult	
Estrogen	Drinking water intake^a^ (mg/person-day)	MOE^b^	Drinking water intake^a^ (mg/person-day)	MOE^c^
Prescribed endogenous estrogens	8.2 × 10^−9^	3,200	1.3 × 10^−8^	1,700
Naturally occurring endogenous estrogens	6.6 × 10^−8^	400	1.1 × 10^−7^	220
Prescribed synthetic estrogens	1.0 × 10^−7^	260	1.6 × 10^−7^	140
Total prescribed estrogens	1.1 × 10^−7^	240	1.7 × 10^−7^	130
Total estrogens from all sources	1.7 × 10^−7^	150	2.8 × 10^−7^	82

a Expressed as E2-eq.

b Compared with a child’s milk intake of 2.6 × 10^−5^ mg/person-day (expressed as E2-eq).

c Compared with an adult dietary intake of 2.3 × 10^−5^ mg/person-day (expressed as E2-eq).

## References

[b1-ehp-118-338] Aerni HR, Kobler B, Rutishauser BV, Wettstein FE, Fischer R, Giger W (2004). Combined biological and chemical assessment of estrogenic activities in wastewater treatment plant effluents. Anal Bioanal Chem.

[b2-ehp-118-338] Aherne GW, Briggs R (1989). The relevance of the presence of certain synthetic steroids in the aquatic environment. J Pharm Pharmacol.

[b3-ehp-118-338] Aksglaede L, Juul A, Leffers H, Skakkebaek NE, Andersson AM (2006). The sensitivity of the child to sex steroids: possible impact of exogenous estrogens. Hum Reprod Update.

[b4-ehp-118-338] Anderson PD, D’Aco VJ, Shanahan P, Chapra SC, Hayes P, Buzby ME (2004). Screening analysis of human pharmaceuticals in U.S. surface waters. Environ Sci Technol.

[b5-ehp-118-338] Andersson AM, Skakkebaek NE (1999). Exposure to exogeneous estrogens in food: possible impact on human development and health. Eur J Endocrinol.

[b6-ehp-118-338] Baronti C, Curini R, D’Ascenzo G, Di Corcia A, Gentili A, Samperi R (2000). Monitoring natural and synthetic estrogens at activated sludge sewage treatment plants and in a receiving river water. Environ Sci Technol.

[b7-ehp-118-338] Belfroid AC, Van der Horst A, Vethaak AD, Schafer AJ, Rijs GBJ, Wegener J (1999). Analysis and occurrence of estrogenic hormones and their glucuronides in surface water and waste water in the Netherlands. Sci Total Environ.

[b8-ehp-118-338] Benotti MJ, Trenholm RA, Vanderford BJ, Holady JC, Stanford BD, Snyder SA (2009). Pharmaceutical and endocrine disrupting compounds in U.S. drinking water. Environ Sci Technol.

[b9-ehp-118-338] Caldwell DJ, Mastrocco F, Hutchinson TH, Länge R, Heijerick D, Janssen C (2008). Derivation of an aquatic predicted no-effect concentration for the synthetic hormone, 17α-ethinyl estradiol. Environ Sci Technol.

[b10-ehp-118-338] Christensen FM (1998). Pharmaceuticals in the environment—a human risk?. Regul Toxicol Pharmacol.

[b11-ehp-118-338] deKleijn MJJ, van der Schouw YT, Wilson PW, Grobbee DE, Jaques PF (2002). Dietary intake of phytoestrogens is associated with a favorable metabolic cardiovascular risk profile in postmenopausal U.S. women: the Framingham study. J Nutr.

[b12-ehp-118-338] Desbrow C, Routledge EJ, Brighty GC, Sumpter JP, Waldock M (1998). Identification of estrogenic chemicals in STW effluent. 1. Chemical fractionation and in vitro biological screening. Environ Sci Technol.

[b13-ehp-118-338] Dey M, Lyttle CR, Pickar JH (2000). Recent insights into the varying activity of estrogens. Maturitas.

[b14-ehp-118-338] Donn J, Mendoza M, Pritchard J (2008a). Pharmaceuticals found in drinking water, affecting wildlife, maybe humans.

[b15-ehp-118-338] Donn J, Mendoza M, Pritchard J (2008b). Fish, wildlife affected by drug contamination in water.

[b16-ehp-118-338] Donn J, Mendoza M, Pritchard J (2008c). No standards to handle pharmaceuticals in water.

[b17-ehp-118-338] Doyle E (2000). Human Safety of Hormone Implants Used to Promote Growth in Cattle.

[b18-ehp-118-338] EPHC (Environment Protection and Heritage Council, National Health and Medical Research Council, and Natural Resource Management Ministerial Council) (2008). Australian Guidelines for Water Recycling: Augmentation of Drinking Water Supplies.

[b19-ehp-118-338] Ferguson PL, Iden CR, McElroy AE, Brownawell BJ (2001). Determination of steroid estrogens in wastewater by immunoaffinity extraction coupled with HPLC-electrospray-MS. Anal Chem.

[b20-ehp-118-338] Food Quality Protection Act of 1996. 1996. Public Law 104-170.

[b21-ehp-118-338] Fritsche S, Steinhart H (1999). Occurrence of hormonally active compounds in food: a review. Eur Food Res Technol.

[b22-ehp-118-338] Hannah R, D’Aco VJ, Anderson PD, Buzby ME, Caldwell DJ, Cunningham VL (2009). Exposure assessment of 17α-ethinyl estradiol in surface waters of the United States and Europe. Environ Toxicol Chem.

[b23-ehp-118-338] Hartmann S, Lacorn M, Steinhart H (1998). Natural occurrence of steroid hormones in food. Food Chem.

[b24-ehp-118-338] Heberer T (2002). Occurrence, fate and removal of pharmaceutical residues in the aquatic environment: a review of recent research data. Toxicol Lett.

[b25-ehp-118-338] Henricks DM, Gray SL, Hoover JLB (1983). Residue levels of endogenous estrogens in beef tissues. J Anim Sci.

[b26-ehp-118-338] Horn-Ross PL, Hoggatt KJ, West DW, Krone MR, Stewart SL, Anton-Culver H (2002). Recent diet and breast cancer risk: the California Teachers Study (USA). Cancer Causes Control.

[b27-ehp-118-338] Houtman CJ, Booij P, van der Valk KM, van Bodegom PM, van den Ende F, Gerritsen AAM (2007). Biomonitoring of estrogenic exposure and identification of responsible compounds in bream from Dutch surface waters. Environ Toxicol Chem.

[b28-ehp-118-338] Howdeshell KL, Furr J, Lambright CR, Wilson VS, Ryan BC, Gray LE (2008). Gestational and lactional exposure to ethinyl estradiol, but not bisphenol A, decreases androgen-dependent reproductive organ weights and epididymal sperm abundance in the male Long Evans hooded rat. Toxicol Sci.

[b29-ehp-118-338] Huang CH, Sedlak DL (2001). Analysis of estrogenic hormones in municipal wastewater effluent and surface water using enzyme-linked immunosorbent assay and gas chromatography/tandem mass spectrometry. Environ Toxicol Chem.

[b30-ehp-118-338] Huang MH, Harrison GG, Mohamed MM, Gornbein JA, Henning SM, Go VL (2000). Assessing the accuracy of a food frequency questionnaire for estimating usual intake of phytoestrogens. Nutr Cancer.

[b31-ehp-118-338] Huggett DB, Foran CM, Brooks BW, Weston J, Peterson B, Marsh KE (2003). Comparison of in vitro and in vivo bioassays for estrogenicity in effluent from North American municipal wastewater facilities. Toxicol Sci.

[b32-ehp-118-338] Klein KO, Martha PM, Blizzard RM, Herbst T, Rogol AD (1996). A longitudinal assessment of hormonal and physical alterations during normal puberty in boys. II. Estrogen levels as determined by an ultrasensitive bioassay. J Clin Endocrinol Metab.

[b33-ehp-118-338] Kolodziej EP, Gray JL, Sedlak DL (2003). Quantification of steroid hormones with pheromonal properties in municipal wastewater effluent. Environ Toxicol Chem.

[b34-ehp-118-338] Kolpin DW, Furlong ET, Meyer MT, Thurman EM, Zaugg SD, Barber LB (2002). Pharmaceuticals, hormones and other organic wastewater contaminants in U.S. streams, 1999–2000: a national reconnaissance. Environ Sci Technol.

[b35-ehp-118-338] Kroes R, Renwick AG, Cheeseman M, Kleiner J, Mangelsdorf I, Piersma A (2004). Structure-based thresholds of toxicological concern (TTC): guidance for application to substances present at low levels in the diet. Food Chem Toxicol.

[b36-ehp-118-338] Kuch HM, Ballschmiter K (2001). Determination of endocrine-disrupting phenolic compounds and estrogens in surface and drinking water by HRGC-(NCI)-MS in the picogram per liter range. Environ Sci Technol.

[b37-ehp-118-338] Laganà A, Bacaloni A, Fago G, Marino A (2000). Trace analysis of estrogenic chemicals in sewage effluent using liquid chromatography combined with tandem mass spectrometry. Rapid Commun Mass Spectrom.

[b38-ehp-118-338] Länge R, Hutchinson TH, Croudace CP, Siegmund F, Schweinfurth H, Hampe P (2001). Effects of the synthetic estrogen 17α-ethinylestradiol on the life-cycle of the fathead minnow (*Pimephales promelas*). Environ Toxicol Chem.

[b39-ehp-118-338] Latendresse JR, Bucci TJ, Olson G, Mellick P, Weis CC, Thorn B (2009). Genistein and ethinyl estradiol dietary exposure in multigenerational and chronic studies include similar proliferative lesions in mammary gland of male Sprague-Dawley rats. Reprod Toxicol.

[b40-ehp-118-338] Masutomi N, Shibutani M, Takagi H, Uneyama C, Hirose M (2004). Dietary influence on the impact of ethinylestradiol-induced alterations in the endocrine/reproductive system with perinatal maternal exposure. Reprod Toxicol.

[b41-ehp-118-338] McCann SE, Freudenheim JL, Marshall JR, Graham S (2003). Risk of human ovarian cancer is related to dietary intake of selected nutrients, phytochemicals and food groups. J Nutr.

[b42-ehp-118-338] Montani C, Penza M, Jeremic M, Biasiotto G, La Sala G, De Felici M (2008). Genistein is an efficient estrogen in the whole-body throughout mouse development. Toxicol Sci.

[b43-ehp-118-338] Mouatassim-Souali A, Tamisier-Karolak S, Perdiz S, Cargouet M, Levi Y (2003). Validation of a quantitative assay using GC/MS for trace determination of free and conjugated estrogens in environmental water samples. J Sep Sci.

[b44-ehp-118-338] Nasu M, Goto M, Kato H, Oshima Y, Tanaka H (2001). Study on endocrine disrupting chemicals in wastewater treatment plants. Water Sci Technol.

[b45-ehp-118-338] Rimoldi G, Christoffel J, Seidlova-Wuttke D, Jarry H, Wuttke W (2007). Effects of chronic genistein treatment in mammary gland, uterus, and vagina. Environ Health Perspect.

[b46-ehp-118-338] Rodgers-Gray TP, Jobling S, Morris S, Kelly C, Kirby S, Janbakhsh A (2000). Long-term temporal changes in the estrogenic composition of treated sewage effluent and its biological effects on fish. Environ Sci Technol.

[b47-ehp-118-338] Salste L, Leskinen P, Virta M, Kronberg L (2007). Determination of estrogens and estrogenic activiity in wastewater effluent by chemical analysis and the bioluminescent yeast assay. Sci Total Environ.

[b48-ehp-118-338] Schwab BW, Hayes EP, Fiori JM, Mastrocco FJ, Roden NM, Cragin D (2005). Human pharmaceuticals in U.S. surface waters: a human health risk assessment. Regul Toxicol Pharmacol.

[b49-ehp-118-338] Spengler P, Körner W, Metzger JW (2001). Substances with estrogenic activity in effluents of sewage treatment plants in southwestern Germany. 1. Chemical analysis. Environ Toxicol Chem.

[b50-ehp-118-338] Ternes TA, Anderson H, Gilberg D, Bonerz M (2002). Determination of estrogens in sludge and sediments by liquid extraction and GC/MS/MS. Anal Chem.

[b51-ehp-118-338] Ternes TA, Kreckel P, Mueller J (1999a). Behaviour and occurrence of estrogens in municipal sewage treatment plants—II. Aerobic batch experiments with activated sludge. Sci Total Environ.

[b52-ehp-118-338] Ternes TA, Stumpf M, Mueller J, Haberer K, Wilken RD, Servos M (1999b). Behavior and occurrence of estrogens in municipal sewage treatment plants—I. Investigations in Germany, Canada and Brazil. Sci Total Environ.

[b53-ehp-118-338] Tsujioka T, Ito S, Ohga A (1992). Female sex steroid residues in the tissues of steers treated with progesterone and oestradiol-17β. Res Vet Sci.

[b54-ehp-118-338] Tyl RW, Myers CB, Marr MC, Castillo NP, Veselica MM, Joiner RL (2008). One-generation reproductive toxicity study of dietary 17β-estradiol (E2; CAS No. 50-28-2) in CD-1^®^ (Swiss) mice. Reprod Toxicol.

[b55-ehp-118-338] U.S. Department of Health and Human Services (2005). Dietary Guidelines for Americans 2005.

[b56-ehp-118-338] U.S. EPA (1997). Exposure Factors Handbook (Final Report).

[b57-ehp-118-338] Webb S, Ternes T, Gibert M, Olejniczak K (2003). Indirect human exposure to pharmaceuticals via drinking water. Toxicol Lett.

[b58-ehp-118-338] WHO (2000). Toxicological Evaluation of Certain Veterinary Drug Residues in Food. WHO Food Additive Series: 43. Estradiol-17β, Progesterone, and Testosterone.

[b59-ehp-118-338] Ying GG, Kookana RS, Ru YJ (2002). Occurrence and fate of hormone steroids in the environment. Environ Int.

